# Molecular and Physiological Regulation of Premature Leaf Senescence in Rice

**DOI:** 10.3390/plants15060869

**Published:** 2026-03-11

**Authors:** Yifan Sun, Jing Wang, Yanchun Yu, Limin Wu, Banpu Ruan

**Affiliations:** College of Life and Environmental Sciences, Zhejiang Provincial Modern Biology and Medicine Industry College, Hangzhou Normal University, Hangzhou 311121, China

**Keywords:** rice, premature leaf senescence, reactive oxygen species, phytohormones, transcription factors

## Abstract

Premature leaf senescence is a major constraint on rice (*Oryza sativa* L.) productivity and yield stability, particularly under increasingly frequent environmental stresses. Unlike developmentally programmed senescence, premature senescence is characterized by early and uncontrolled activation of senescence pathways, leading to accelerated chlorophyll degradation, oxidative damage, impaired photosynthesis, and reduced grain filling. Recent studies have revealed that premature senescence in rice is governed by a complex regulatory network integrating reactive oxygen species (ROS) homeostasis, phytohormone signaling, transcriptional regulation, and environmental cues. Central signaling hubs involving abscisic acid, ethylene, jasmonic acid, cytokinins, and gibberellins interact extensively with ROS metabolism to fine-tune senescence onset and progression. These upstream signals converge on key transcription factor families, particularly NAC and WRKY proteins, which directly regulate senescence-associated genes responsible for chloroplast dismantling, nutrient remobilization, and programmed cell death. Moreover, abiotic stresses such as drought, salinity, temperature extremes, and nitrogen deficiency commonly trigger premature senescence through a shared ABA–ROS signaling module. This review systematically summarizes recent advances in the physiological characteristics, molecular mechanisms, and environmental regulation of premature leaf senescence in rice, and discusses emerging genetic and agronomic strategies to delay senescence. A deeper understanding of senescence regulatory networks will facilitate the development of rice cultivars with prolonged photosynthetic duration, improved stress resilience, and enhanced yield stability under changing climatic conditions.

## 1. Introduction

The growing global population presents a significant challenge to food security. The current food production system relies heavily on a few staple crops, such as wheat (*Triticum aestivum* L.), rice, and maize (*Zea mays* L.), which makes it difficult to meet the increasing demand for food in the future. Despite continuous improvements in agricultural productivity, over 820 million people worldwide still face food insecurity, and at least 2 billion suffer from various forms of malnutrition [1]. In rice production, premature leaf senescence induced by extreme climate events and improper agricultural practices has become a critical factor limiting rice yield and grain quality. Therefore, understanding the regulatory mechanisms of premature senescence in rice is crucial for ensuring global food security.

**Figure 1:**
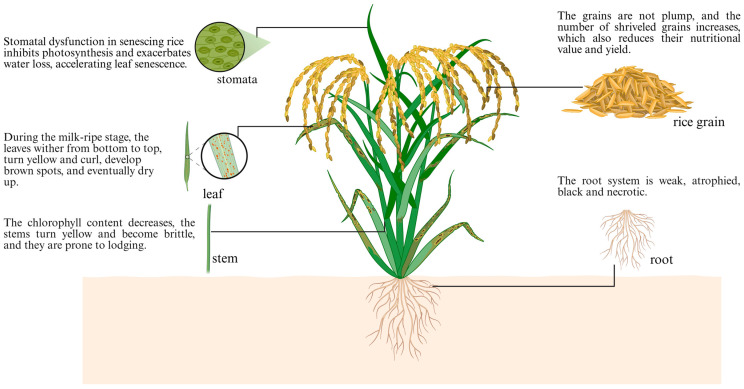
Physiological changes associated with premature senescence in rice.

Leaf senescence is the final stage of leaf development, a genetically programmed process that remobilizes nutrients from senescing leaves to developing sink organs, such as grains, stems, and roots, enabling resource recycling [2,3]. This process is marked by leaf chlorosis, accompanied by a decline in photosynthetic capacity, and involves an active regulatory shift from nutrient absorption to nutrient recycling [4,5]. A key distinction in rice is between natural and premature senescence: natural senescence contributes to grain filling by ensuring efficient nutrient redistribution, thereby enhancing yield; in contrast, premature senescence, caused by premature activation of the senescence program, disrupts the transport of photosynthates, reduces seed-setting rate, and ultimately leads to significant losses in both yield and rice quality [5,6,7] (Figure 1). Therefore, understanding the regulatory mechanisms of premature senescence and identifying strategies to prolong leaf functional lifespan are central objectives in rice improvement.

Premature senescence is a multifactorial trait arising from the complex interplay between genetic background, environmental signals, and endogenous physiological states [8,9,10]. Under abiotic stress conditions, the disruption of ROS homeostasis in crops such as rice induces oxidative damage to biomacromolecules, promotes membrane lipid peroxidation, and increases membrane permeability. These changes accelerate cellular dysfunction and ultimately lead to cell death via the oxidative stress pathway [11]. In parallel, senescence-promoting hormones such as abscisic acid (ABA) [12,13], ethylene (ETH) [14,15] and jasmonic acid (JA) [16] are typically upregulated, whereas senescence-delaying hormones, most notably cytokinins (CTKs), decline in abundance [17]. These hormonal shifts collectively reinforce senescence signaling cascades and promote the premature activation of senescence programs.

At the transcriptional level, several transcription factor families, including NAC (NAM, ATAF1/2, CUC2) and WRKY, play pivotal roles in integrating ROS and hormone-derived signals and in orchestrating the expression of senescence-associated genes. Through precise transcriptional control, these regulators drive key physiological processes such as chlorophyll degradation, protein turnover, and nutrient remobilization. Among them, WRKY transcription factors and ABA-responsive cis-elements have been repeatedly identified as central components of senescence regulatory networks, highlighting their potential as molecular entry points for targeted modulation of premature senescence [18,19].

In addition to the conserved molecular regulatory pathways, the genetic background and ecological adaptability of rice varieties themselves profoundly influence the occurrence of premature senescence. A noteworthy yet often overlooked aspect is the diversity of rice seasonal ecotypes. Based on their responses to temperature and photoperiod, rice is classified into three types: early-season, medium-season, and late-season, each exhibiting significant differences in premature senescence susceptibility [20,21,22,23]. Early-season rice, characterized by a short growth duration and weak photoperiod sensitivity, coupled with a high proportion of indica varieties, shows natural variations in the *OsSGR* gene promoter that accelerate chlorophyll degradation [21]; this makes it more prone to photo-oxidative damage and premature senescence under conditions of low temperature and high light intensity [20]. For medium-season rice, the extent of premature senescence varies between indica and japonica types: indica medium-season rice is more susceptible, whereas japonica medium-season rice exhibits a lower incidence of premature senescence due to its stronger tolerance to photo-oxidative damage [20].

In contrast, late-season rice undergoes grain filling and maturation during the relatively mild conditions of autumn. This environment presents a stark contrast to the heat stress during grain filling that disrupts the expression of grain-filling-related genes and induces premature senescence [22]. Although environmental stress is generally lower for late-season rice, varietal characteristics remain crucial for determining its overall resilience and productivity. Late-season rice comprises a higher proportion of japonica varieties, which possess enhanced tolerance to abiotic stresses [23]. This inherent tolerance safeguards against sporadic or mild stress, thereby enhancing yield stability and further minimizing the risk of premature senescence.

These differences among ecotypes indicate that the regulation of premature senescence involves not only conserved molecular pathways but also fine-tuning by variety-specific genetic variations, providing a theoretical basis for breeding premature senescence-resistant rice varieties adapted to different ecological regions.

This review synthesizes recent advances in the physiological, molecular, and genetic regulation of rice leaf senescence, with particular emphasis on mechanisms underlying premature senescence. By integrating insights from functional genomics, hormone biology, redox regulation, and transcriptional networks, we aim to provide a comprehensive framework for understanding senescence control in rice. Such knowledge is essential for the development of rice cultivars combining high yield potential with enhanced yield stability under increasingly variable environmental conditions [24]. Furthermore, emerging breeding strategies that integrate molecular breeding, genetic engineering, and senescence-specific regulatory elements offer promising avenues to delay leaf senescence, for example by maintaining CTK homeostasis. Looking forward, the application of artificial intelligence and Internet of Things-based technologies for high-throughput, non-destructive phenotyping is expected to enable precise monitoring and prediction of premature senescence in the field. Coupled with systems biology approaches, these advances will facilitate the rational design of senescence traits, providing new opportunities to address global climate change and ensure sustainable food security [25].

## 2. Early Physiological Characteristics and Signal Perception of Premature Senescence

### 2.1. Perception of Senescence Signals by Chloroplasts

Chloroplasts not only execute senescence but also perceive its signals at an early stage. At the onset of premature senescence, impairment of the photosynthetic electron transport chain leads to ROS production, which then act as early signaling molecules to activate downstream senescence-associated genes [26]. Concurrently, the accumulation of photosynthetic products such as sugars can serve as metabolic signals, relayed to the nucleus through sensors like hexokinase (*OsHXK1*), thereby initiating the senescence program [27].

Chlorophyll degradation proceeds through a highly conserved, multi-step enzymatic pathway. Initially, chlorophyll *b* (Chl *b*) is reduced to chlorophyll *a* (Chl *a*) by the Chl *b* reductase complex composed of NON-YELLOW COLORING 1 (*NYC1*) and NYC1-LIKE (*NOL*) proteins [28]. The subsequent core steps involve porphyrin ring opening, during which Chl *a* is converted into pheophorbide *a* (Pheide *a*) following magnesium dechelation. Pheide *a* is then cleaved by the key enzyme pheophorbide *a* oxygenase (PAO) to produce red chlorophyll catabolite (RCC), which is subsequently reduced by red chlorophyll catabolite reductase (RCCR) to form the colorless primary fluorescent chlorophyll catabolite (pFCC) [29]. PAO functions as the rate-limiting enzyme in this pathway and is indispensable for orderly chlorophyll breakdown. Mutant studies have confirmed the importance of this regulatory node, as disruption of PAO function in mutants such as maize *lls1* or Arabidopsis (*Arabidopsis thaliana*) *acd1* leads to the accumulation of phototoxic pheide a, which triggers ROS bursts and cell death upon light exposure [30]. This mechanism is directly relevant to premature senescence, as PAO dysfunction accelerates leaf degradation and contributes to early senescence.

The execution of this degradation pathway is tightly regulated at multiple genetic levels, and culminates in the action of key enzymatic components. The STAY-GREEN (*SGR*) protein, a central enzyme in this process, plays a pivotal “executor” role. SGR is specifically induced during senescence and promotes chlorophyll degradation by directly interacting with light-harvesting complex I and II (LHCPI/II), thereby destabilizing pigment–protein complexes and exposing chlorophyll molecules to downstream catabolic enzymes [31]. Consequently, *sgr* loss-of-function mutants exhibit a non-functional stay-green phenotype, characterized by retained green coloration alongside declining photosynthesis and delayed chloroplast dismantling [4]. Mutations in chlorophyll degradation genes other than *SGR*, such as *nyc3*, also lead to a stay-green phenotype, albeit weaker than that of *sgr* mutants. Moreover, chlorophyll retention in *nyc3 sgr* double mutants is similar to that of *sgr* single mutants, suggesting that SGR acts downstream of or in concert with NYC3 in the same regulatory framework [31]. *NYC3* encodes a plastid-localized protein belonging to the α/β hydrolase family. The protein contains an esterase/lipase domain and is thought to participate in the early steps of chlorophyll degradation, although its precise function remains to be further investigated [31]. Beyond these loss-of-function effects, variation in SGR expression levels also influences senescence timing. Promoter variations in the *SGR* gene lead to earlier activation of the senescence program in certain rice varieties, resulting in higher gene expression, faster chlorophyll degradation, and ultimately premature senescence [21].

Upstream of this regulatory hierarchy, multiple transcription factors and kinases integrate endogenous and environmental cues to coordinate the expression of chlorophyll degradation-associated genes. For instance, *OsDOF24* functions as a negative regulator of senescence. Gain-of-function mutants (*Osdof24-D*) delay senescence by directly binding to and repressing the promoter of the JA biosynthetic gene *OsAOS1*, thereby reducing endogenous JA levels and downregulating the expression of degradation-related genes such as *NYC1*, *NYC3*, and *SGR* [32]. In contrast, *OsWRKY5* and *OsNAC2* serve as central positive regulators of senescence. *OsWRKY5* indirectly promotes senescence by upregulating senescence-associated *NAC* genes (*OsNAP*) and ABA biosynthesis genes such as *OsNCED3* [33]. *OsNAC2* directly binds to the promoters of *OsSGR* and *OsNYC3*, activating their transcription, while simultaneously reinforcing senescence through a positive feedback loop involving ABA metabolism [34]. These two transcription factors contribute to premature senescence through distinct pathways: OsWRKY5 is primarily induced by aging and dark treatment, and its overexpression accelerates leaf yellowing [33]; OsNAC2 is rapidly activated by abiotic stresses via ABA signaling, directly initiating chlorophyll degradation genes and accelerating premature senescence [13,34]. Together, they integrate developmental and stress signals to trigger premature senescence. In addition to the transcriptional regulators discussed above, multiple kinases also play key roles in integrating environmental signals to regulate chlorophyll degradation and the senescence process. For example, the senescence-induced receptor-like kinase *OsSRLK* is highly expressed during dark-induced senescence and is required for proper chlorophyll degradation [35]. Mutation of *OsSRLK* impairs chlorophyll degradation and preserves chloroplast ultrastructure, indicating that it is indispensable for chloroplast dismantling [35]. Transcriptomic analysis reveals that *OsSRLK* functions by modulating phytohormone signaling pathways, particularly those involving ABA, ethylene, and jasmonic acid, thereby influencing the progression of leaf senescence [35].

Chlorophyll degradation is closely coordinated with the dismantling of chloroplast ultrastructure. During senescence, thylakoid membrane stacking (grana) gradually disintegrates, accompanied by the degradation of photosystem I and II proteins [36]. Stay-green mutants illustrate the interdependence of these processes. In stay-green mutants such as *sgr* and *nyc1*, enhanced stability of LHCII delays thylakoid degradation, underscoring the importance of intact chlorophyll–protein complexes for maintaining chloroplast structure [37]. In contrast, this process is markedly accelerated in premature senescence mutants. The physiological changes in these mutants phenocopy those observed in stress-induced premature senescence. For example, mutation of the C2H2-type zinc finger protein gene *LS1* leads to excessive accumulation of ROS (H_2_O_2_) and malondialdehyde (MDA), increased superoxide dismutase (SOD) activity, reduced catalase (CAT) activity, severe DNA degradation, and enhanced programmed cell death (PCD), thereby accelerating chloroplast disintegration [38]. Similarly, loss of function of the photosystem II subunit *OsPsbS1* compromises photoprotective capacity, resulting in excessive ROS accumulation that triggers chloroplast degradation and premature senescence [39]. Together, these examples demonstrate that disrupting protective or regulatory mechanisms can directly trigger premature senescence by accelerating chloroplast dismantling [38,39].

Collectively, chloroplast and chlorophyll degradation during rice leaf senescence constitute a highly programmed process initiated by upstream signals such as JA, ABA, and ROS, integrated by core transcriptional regulators (including *OsDOF24*, *OsWRKY5*, and *OsNAC2*), and ultimately executed by key effector components such as SGR, NYC1, and PAO. In premature senescence, this regulatory network is initiated ahead of schedule, often triggered by stress-induced ROS bursts [13,26] or hormonal imbalances [13]. As a result, senescence-associated genes are activated early, and chloroplast degradation accelerates before grain filling is complete.

### 2.2. Loss of Cellular Structure and Membrane Integrity

The decline in total protein content not only weakens photosynthetic efficiency in rice but also suppresses nutrient metabolism and energy conversion pathways. In premature senescence, this decline is accelerated, hastening the loss of photosynthetic capacity. As protein biosynthesis slows and proteolysis accelerates, plant growth becomes increasingly constrained, leading to reduced biomass accumulation and ultimately lower yield [40]. The sustained loss of photosynthesis-related proteins in leaves undermines the stability of photosynthetic function, exerting systemic effects on overall plant growth and development [27,41]. Previous studies have indicated that the hexokinase gene *OsHXK1* functions as a positive regulator of leaf senescence in rice. As a bifunctional enzyme involved in both glycolysis and sugar signaling, *OsHXK1* is thought to contribute to the metabolic regulation that influences senescence-associated gene expression, likely through glucose accumulation and ROS production [27].

Membrane lipid peroxidation is a central mechanism underlying the loss of membrane integrity during premature senescence. This process is typically assessed by measuring MDA content, one of the principal end products of lipid peroxidation and a widely used indicator of membrane oxidative damage [42]. During premature senescence in rice, excessive accumulation of ROS progressively degrades unsaturated fatty acids in the cell membrane, triggering lipid peroxidation and generating large quantities of MDA [43]. As a by-product of lipid peroxidation, MDA accumulation serves as a reliable indicator of oxidative damage to the cell membrane [44]. Importantly, MDA is not merely a passive marker but an active mediator of cellular damage: it readily forms cross-links with macromolecules such as proteins and nucleic acids, altering their conformations and impairing their biological functions. In addition, MDA can loosen intermolecular bridges within cellulose and inhibit protein biosynthesis [45].

A self-amplifying feedback loop operates between ROS and MDA. ROS exacerbate lipid peroxidation and promote MDA production, while MDA in turn perturbs cellular redox status, creating conditions conducive to further ROS accumulation [26]. This mutually reinforcing cycle continuously accelerates leaf senescence. In rice, overexpression of the senescence-associated hexokinase gene *OsHXK1* leads to a synchronous increase in both ROS and MDA levels in leaves [27]. This correlation suggests a potential link between OsHXK1-mediated signaling and the progression of membrane lipid peroxidation, which may amplify oxidative damage and accelerate the senescence cascade. Further investigation is required to determine whether *OsHXK1* directly modulates lipid peroxidation or if the elevated MDA is a consequence of increased ROS production. The functional consequence of lipid peroxidation is manifested as altered membrane permeability and disrupted membrane integrity. The plasma membrane is a fundamental structural component of plant cells, playing essential roles in transmembrane transport and the maintenance of intracellular homeostasis [46]. Membrane injury impairs ion channel function and alters membrane permeability, disrupting ionic homeostasis and promoting senescence progression. As membrane integrity deteriorates, cells lose the ability to maintain internal–external equilibrium, resulting in restricted plant growth and aggravated senescence [45,47,48,49]. Under conditions that induce premature senescence, such as nitrogen deficiency [47] or genetic mutation [45], oxidative damage in leaves is intensified and occurs earlier, as evidenced by significantly elevated ROS and MDA levels [45,47], ultimately leading to accelerated membrane deterioration [47,48]. Leaf senescence in rice is tightly associated with progressive damage to the cell membrane system, a process in which membrane lipid peroxidation plays a critical role. ROS attack polyunsaturated fatty acids in membrane phospholipids and induce a chain reaction, and products such as MDA disrupt the ordered structure of membrane lipids and oxidize membrane proteins [50]. As premature senescence advances, this oxidative damage intensifies, often becoming irreversible and directly compromising multiple physiological processes. Therefore, suppressing lipid peroxidation and maintaining membrane structural stability are critical strategies for delaying premature senescence in rice [51].

The alteration in membrane permeability is not an isolated event, but a hallmark of severe dysfunction of ion channels and transporters. This is followed by the collapse of intracellular ion homeostasis of ions such as K^+^ and Ca^2+^, and the aberrance of Ca^2+^ signaling can in turn activate NADPH oxidase to generate more ROS. Consequently, a self-amplifying vicious cycle is established: oxidative damage elevates membrane permeability, which in turn disrupts ionic homeostasis; this ionic imbalance further exacerbates oxidative injury. This relentless cycle ultimately accelerates the comprehensive disintegration of the membrane system [45]. In premature senescence, stress-induced ROS accumulation [13] can trigger this vicious cycle at an early stage, exacerbating membrane damage before grain filling is complete. This vicious cycle is coupled with the active genetic program of senescence. For instance, *OsNAP*, a core senescence-regulating transcription factor, actively remodels intracellular ion distribution and the cellular microenvironment by directly activating downstream target genes, including those involved in ion transport, thereby driving the senescence process in a systemic manner [52]. *OsNAP* expression is upregulated under stress conditions and directly activates chlorophyll degradation genes such as *SGR*, *NYC1*, and *NYC3* [52], thereby contributing to the early onset of chloroplast dismantling and membrane dysfunction in premature senescence.

As leaf senescence progresses, membrane damage spreads from the plasma membrane to all organellar membranes. Damage to the thylakoid membranes of chloroplasts results in a decrease in the efficiency of photosynthetic electron transport, such as the Fv/Fm ratio [53]; the loss of mitochondrial membrane integrity triggers the uncoupling of oxidative phosphorylation and an energy metabolism crisis [54]. Ultimately, the altered permeability of the vacuolar membrane leads to the release of hydrolases, which accelerates the degradation of macromolecules and drives cells into programmed cell death. In premature senescence mutants such as *ls1* [36] and *Ospsbs1* [37], chloroplast degradation is accelerated, leading to a rapid decline in photosynthetic function during the critical grain-filling period.

Thus, increased MDA content serves not only as a diagnostic marker of premature senescence but also as a direct indicator of cellular oxidative damage [45]. Reducing MDA accumulation may contribute to delaying premature senescence, alleviating membrane damage, and maintaining normal rice growth and physiological function [34].

## 3. Core Signaling Pathways of Premature Senescence

The onset of premature senescence in rice is governed by complex signaling networks that integrate internal regulatory mechanisms and external environmental factors [13,48]. ROS, including superoxide anion (O_2_^−^), hydrogen peroxide (H_2_O_2_), and hydroxyl radical (-OH), are generated through multiple metabolic pathways, such as mitochondrial respiration and photosynthetic electron transport in chloroplasts [55]. During premature senescence, ROS metabolism extends beyond a simple balance between production and scavenging, functioning instead as a dynamic signaling network [56]. Specific signals associated with premature senescence—such as ABA accumulation, pathogen infection, or developmental abnormalities—activate plasma membrane-localized NADPH oxidases (*Rbohs*), leading to rapid, localized production of H_2_O_2_ that ensures signaling specificity [57]. This locally generated H_2_O_2_ subsequently oxidizes cysteine residues in target proteins in a reversible manner, including MAPK cascade kinases and senescence-associated transcription factors (certain *NAC* or *WRKY* proteins), thereby converting chemical signals into biological instructions that regulate leaf senescence [58]. The specificity and reversibility of H_2_O_2_ signaling are maintained through the interplay between H_2_O_2_-generating and scavenging systems. Peroxiredoxins (Prxs) continuously eliminate basal levels of H_2_O_2_ to prevent non-specific oxidative damage [59]. However, when premature senescence signals trigger substantial H_2_O_2_ production, Prx activity is transiently suppressed, allowing H_2_O_2_ to overcome the local antioxidant barrier and interact with signaling targets. Subsequently, thioredoxin (Trx), as the core of the reduction system, helps restore the function of oxidized Prxs and target proteins, thereby achieving precise signal termination [59,60]. Typically, environmental stress acts as an initial signal, inducing phytohormone crosstalk and ROS metabolic imbalance, which together form a central regulatory hub. These signals are subsequently integrated and transmitted by transcription factor networks, leading to the systemic activation of senescence-associated physiological processes and gene expression programs.

### 3.1. Imbalance in ROS Metabolism

#### 3.1.1. ROS Production and Its Detrimental Effects

One of the most prominent biochemical features of premature senescence in rice is the excessive accumulation of ROS. Under normal metabolic conditions, ROS are maintained at low levels and function as signaling molecules involved in various physiological processes. However, when plants are exposed to environmental or developmental stress, the balance between ROS production and scavenging is disrupted, leading to ROS overaccumulation and the onset of oxidative stress. Excessive ROS cause oxidative damage to cellular components, including membranes, proteins, lipids, and DNA, thereby accelerating the senescence process [10].

During premature senescence in rice, ROS accumulation triggers a cascade of detrimental effects. First, excessive ROS disrupt the lipid bilayer structure of cellular membranes, altering membrane fluidity and impairing membrane function. Such structural damage compromises intracellular material transport and membrane integrity. Second, ROS-induced oxidative modification of proteins leads to structural and functional impairment. In rice leaves, excessive ROS accumulation particularly damages chloroplast membranes, reduces the activity of photosynthesis-related enzymes, and ultimately lowers photosynthetic efficiency. In parallel, oxidative damage to DNA can induce mutations, further promoting cellular senescence and programmed cell death [10,61]. As premature senescence progresses, the cellular antioxidant system becomes increasingly imbalanced and incapable of efficiently removing accumulated ROS, resulting in progressively aggravated oxidative damage.

#### 3.1.2. Responses of the Antioxidant System

The plant antioxidant enzyme system constitutes the primary defense against ROS accumulation. SOD acts as the first line of defense by catalyzing the conversion of O_2_^−^ into H_2_O_2_, which is subsequently detoxified into water and oxygen by CAT and Peroxidase (POD) [55]. During premature senescence in rice, antioxidant enzyme activities typically exhibit a characteristic dynamic pattern, initially increasing and subsequently declining. At early stages, the activities of SOD, POD, and related enzymes are compensatorily elevated to counteract ROS bursts. However, with prolonged stress exposure or advancing senescence, the antioxidant system becomes exhausted, enzyme activities decline sharply, and ROS can no longer be effectively scavenged. This leads to intensified oxidative damage and establishes a vicious cycle that accelerates senescence progression [44]. In addition, phytohormones such as ABA can modulate ROS levels by regulating the expression of antioxidant enzyme genes, highlighting the tight interconnection between hormone signaling and ROS homeostasis.

Beyond its role as a causative agent of oxidative damage, ROS, particularly H_2_O_2_, function as early signaling molecules that activate downstream senescence programs. As detailed in the following section, H_2_O_2_ can oxidize cysteine residues in MAPK cascade kinases and senescence-associated transcription factors such as *NAC* and *WRKY* proteins, thereby converting oxidative signals into specific biological instructions that initiate the expression of senescence-associated genes [58].

### 3.2. Hormonal Regulation and Signal Crosstalk

**Figure 2:**
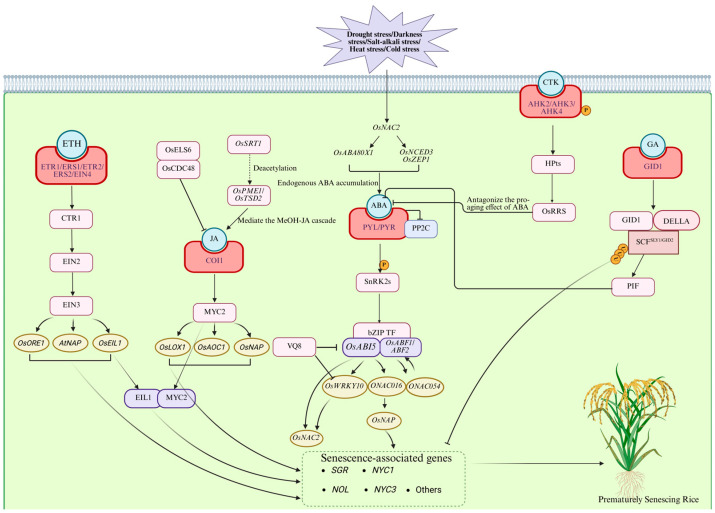
Hormonal signaling regulation and crosstalk underlying premature senescence in rice. This schematic illustrates the signaling pathways and extensive crosstalk among five major phytohormones—ETH, JA, ABA, CTK, GA—in rice under abiotic stresses such as drought, darkness, and high or low temperature. Stress signals initially induce the expression of hormone metabolism-related genes, leading to the accumulation of senescence-promoting hormones (ABA, ETH, and JA) and/or the modulation of senescence-delaying hormones (CTK and GA). Subsequently, hormonal signals are transduced through the ETH (ETR/ERS–CTR1–EIN2–EIN3), JA (COI1–JAZ–MYC2), ABA (PYL–PP2C–SnRK2s), CTK (AHK–HPs–OsRRs), and GA (GID1–DELLA) signaling pathways, respectively. These pathways are extensively interconnected through protein–protein interactions and antagonistic or synergistic signaling mechanisms. Ultimately, they converge on key transcription factors such as OsNAP and OsNACs, which activate downstream senescence-associated genes, including chlorophyll degradation genes and senescence marker genes, thereby driving the progression of premature leaf senescence in rice. Solid arrows indicate direct signal transduction, lines with a vertical bar at the end indicate repression, and dashed arrows indicate indirect regulation through intermediate metabolites or multi-step cascades.

Phytohormones play indispensable roles in regulating premature senescence in rice and act as central signaling molecules in this process. Interactions among different hormones form a highly complex and finely tuned regulatory network (Figure 2). Among them, ABA, ETH, and JA function predominantly as senescence-promoting hormones, whereas CTKs, brassinosteroids (BRs), and gibberellins (GAs) mainly exert antagonistic, senescence-delaying effects [62].

#### 3.2.1. ABA

ABA is a sesquiterpenoid phytohormone that not only regulates plant responses to biotic and abiotic stresses but also plays an important regulatory role in multiple aspects of plant growth and development, highlighting its importance as a key player in plant adaptation processes [63]. Elevated ABA levels during senescence induce the expression of senescence-associated transcription factors, promote cellular component degradation, and accelerate leaf yellowing. ABA upregulates Chl *b* reductase genes *NYC1* and *NOL*, thereby enhancing chlorophyll degradation and nutrient remobilization [64], ultimately leading to leaf senescence and abscission. Exogenous ABA treatment induces premature senescence; studies on detached rice leaves have shown that ABA application significantly increases H_2_O_2_ and MDA contents while reducing chlorophyll levels, indicating aggravated oxidative damage and accelerated senescence [62].

Under stress conditions, ABA rapidly accumulates and promotes senescence partly by inducing ETH production. This process involves activation of SNF1-related protein kinases 2 (SnRK2s) in an ETH-independent manner, followed by phosphorylation of ABA response element-binding factors (ABFs). Phosphorylated ABFs, together with *RAV1*, upregulate senescence-associated genes. Upon perception of ABA by PYR/PYL/RCAR receptors [12], protein phosphatase 2Cs (PP2Cs) are inhibited, leading to SnRK2 activation. Activated SnRK2s phosphorylate downstream basic leucine zipper (*bZIP*) transcription factors, enhancing their transcriptional activity. These activated transcription factors directly bind to promoters of senescence-associated genes, such as *OsNAP* and *OsSGR*, upregulating their expression and ultimately promoting chlorophyll degradation and cell death [17,65]. *OsNAP* links ABA signaling to leaf senescence by finely regulating ABA biosynthesis and directly targeting SAGs. *WRKY* transcription factors act as either activators or repressors within ABA signaling; for example, *LtWRKY21* functions as an activator of ABA-regulated gene expression [66]. This functional duality is further exemplified in the fine-tuning of leaf senescence in rice. Recent research has revealed that *WRKY10* interacts with the ABA-responsive element-binding factors *ABF1/2* and the VQ motif-containing protein *VQ8*, forming a regulatory unit described in the original study as an “accelerator-brake module,” which is essential for the precise regulation of dark- and ABA-induced senescence. Within this system, *WRKY10* acts as a positive regulator that promotes ABA- and dark-induced senescence by directly controlling the expression of multiple senescence-associated genes, whereas *VQ8* serves as a suppressor of *WRKY10* and negatively modulates *WRKY10*-mediated senescence. Moreover, *VQ8* can repress the transcriptional activity of *ABF1* and *ABF2.* ABA, methyl jasmonate, and H_2_O_2_ accelerate *WRKY10*-driven senescence, whereas ammonium nitrate and dithiothreitol delay it. This module integrates multiple senescence signals to ensure the orderly progression of leaf senescence, while *VQ8* functionally acts as a “brake” that also suppresses *ABF1*- or *ABF2*-induced cell necrosis, thereby preventing uncontrolled cell death [67].

#### 3.2.2. ETH

ETH is a gaseous phytohormone that plays a pivotal role in determining the extent of leaf senescence. Increased ETH levels in senescing leaves are accompanied by upregulation of ETH biosynthesis genes, such as those encoding ACC synthase and ACC oxidase, as well as signaling components. Transcriptomic analyses indicate that approximately 25% of ETH-related genes are significantly upregulated during senescence, consistent with increased ETH accumulation in aging leaves [68,69]. Exogenous ETH treatment accelerates chlorophyll and protein degradation in rice leaves, thereby promoting senescence, whereas inhibitors of ETH biosynthesis or signaling delay senescence, further confirming the positive regulatory role of ETH [70,71].

ETH is perceived by five receptors localized to the endoplasmic reticulum membrane: ETHYLENE RECEPTOR 1 (*ETR1*), ETHYLENE RESPONSE SENSOR 1 (*ERS1*), and the subfamily II receptors *ETR2*, *ERS2*, and ETHYLENE INSENSITIVE 4 (*EIN4*) [72]. Downstream signaling is sequentially transmitted through CONSTITUTIVE TRIPLE RESPONSE 1 (*CTR1*), ETHYLENE INSENSITIVE 2 (*EIN2*), ETHYLENE INSENSITIVE 3 (*EIN3*)/EIN3-LIKE 1 (*EIL1*), and ETHYLENE RESPONSE FACTORS (*ERFs*) [73]. ETH signaling is closely linked to *NAC* transcription factor cascades; for instance, *ORE1* (*ANAC092*) is positively regulated by *EIN2* and negatively regulated by *miR164*, resulting in increased *ORE1* expression and promotion of leaf senescence [74]. The downstream signaling component *EIN3* directly activates *ORE1* and *AtNAP*, thereby accelerating senescence [75]. In addition to their well-defined functions in Arabidopsis and rice, the senescence-promoting role of *NAC* transcription factors—core components of this regulatory network—has also been confirmed in other plant species. For example, in sunflower (*Helianthus annuus*), the ortholog of *ORE1*/*AtNAP*, *HaNAC1*, has been demonstrated to accelerate leaf senescence when overexpressed in Arabidopsis [76], supporting the conservation of this pathway across the plant kingdom. It should be noted, however, that the intricate trifurcate feed-forward loop specifically involving *ORE1*, *miR164*, and *AtNAP* has been characterized primarily in Arabidopsis, and its conservation across a broad range of species awaits further systematic validation.

#### 3.2.3. JA

JA is a lipid-derived hormone synthesized from α-linolenic acid in chloroplast membranes and plays essential roles in plant growth and development, including regulation of secondary metabolism, leaf senescence and abscission, and fruit ripening [77]. JA participates in defense responses and stress adaptation in rice by modulating the synthesis and release of volatile compounds. Research has demonstrated that the JA signaling pathway plays a central regulatory role in the biosynthesis of both herbivore-induced and constitutive volatiles in rice [78,79]. During natural senescence or under stress conditions, endogenous JA levels increase markedly [80]. Exogenous application of methyl jasmonate (MeJA) to rice leaves significantly reduces chlorophyll content, impairs light energy capture and conversion, disrupts photosynthesis, and thereby promotes premature senescence. In rice, *OsNAP* positively regulates leaf senescence through the JA pathway; overexpression of *OsNAP* elevates JA levels and upregulates JA biosynthesis genes such as *LOX2* and *AOC1*, further accelerating senescence [81].

#### 3.2.4. Synergistic Regulation by ETH and JA

The synergistic interplay between ETH and JA represents a key hormonal interaction during premature senescence in rice. At the onset of premature senescence, the ETH signaling transcription factor *OsEIL1* physically interacts with the JA signaling regulator *OsMYC2* to form a protein complex [16]. This complex synergistically binds to the promoters of the master senescence regulator *OsNAP* and the key chlorophyll degradation gene *OsSGR*, thereby significantly enhancing their transcriptional activity and collectively promoting the progression of premature senescence [82].

#### 3.2.5. CTKs

CTKs are N^6^-substituted adenine derivatives and represent key senescence-delaying hormones. CTKs suppress leaf senescence by maintaining chlorophyll content, sustaining photosynthetic efficiency, promoting nutrient redistribution, and stimulating cell division and shoot growth. During leaf senescence, endogenous CTK levels decline significantly [83,84]. Increasing CTK levels through exogenous application or genetic engineering effectively delays leaf senescence in multiple plant species. For instance, treatment with 0.1–10.0 μg/L of the novel CTK compound forchlorfenuron (KT30) slows the degradation of chlorophyll, proteins, and soluble sugars while enhancing antioxidant enzyme activities (CAT and SOD), thereby delaying senescence in detached leaves [85].

CTK signaling is transduced through a histidine phosphorelay system that activates CTK response regulators (*OsRRs*) [86]. *OsRRs* function as transcriptional repressors or activators involved in phytohormone crosstalk. For instance, *OsRR26* responds to both cytokinin and ABA and participates in ABA-mediated processes such as ROS compartmentalization [87]. Additionally, the ABA catabolic gene *OsABA8ox3* has been characterized for its role in drought stress response [88], suggesting that ABA homeostasis is tightly controlled at multiple levels. In addition, CTK signaling enhances antioxidant system activity [85], alleviating ABA-induced ROS accumulation and delaying senescence at the physiological level [89]. Collectively, CTKs play a critical role in maintaining prolonged leaf greenness in rice, particularly under unfavorable conditions such as drought stress and low nitrogen availability, where exogenous CTK supplementation effectively delays premature senescence [85].

#### 3.2.6. GAs

GAs are diterpenoid phytohormones involved in regulating various physiological processes in plants [90], and their biosynthesis and signaling pathways have been extensively studied [91,92]. In rice leaves, GA functions in a stage-specific manner during senescence, promoting senescence initiation at early stages while exerting delaying effects at later stages through feedback regulation. GA-deficient or senescence-related mutants such as *esd1* exhibit premature senescence phenotypes, underscoring the importance of GA in maintaining normal leaf lifespan [93]. Exogenous GA application delays premature senescence, as evidenced by reduced MDA accumulation in both wild-type Nipponbare (NIP) and the premature senescence mutant *esd1*, with a more pronounced effect in *esd1*, indicating that GA signaling negatively regulates premature senescence in rice [93]. Hormone treatments of wild-type rice and the premature senescence mutant *esd1* with GA_3_ and low concentrations of KT30 both reduce MDA accumulation, with more pronounced effects observed in *esd1* [93].

At the molecular level, GA binds to its receptor GID1 to form a complex that promotes recognition and degradation of DELLA proteins by the SCF^GID2/SLY1^ ubiquitin ligase complex [94]. Removal of DELLA proteins alleviates their repression of senescence-promoting transcription factors, thereby downregulating the expression of chlorophyll degradation enzymes and proteases. Simultaneously, DELLA degradation releases growth-promoting transcription factors such as *PIFs*, antagonizes ABA biosynthesis and signaling, and enhances antioxidant system activity to mitigate ROS accumulation, collectively contributing to delayed leaf senescence [95].

### 3.3. Gene Regulation and Transcription Factors

The molecular mechanisms underlying premature senescence in rice are highly complex and finely coordinated, involving numerous key genes and transcription factors (TFs) (Table 1). With advances in molecular biology, an increasing number of senescence-associated genes have been identified. These genes regulate the onset and progression of premature senescence by modulating core biological processes such as carbohydrate metabolism, nitrogen utilization, and protein degradation [73].

*OsSWEET1b* is a hexose transporter and a key member of the SWEET family involved in sugar metabolism regulation, and it has been demonstrated to play an important role in rice premature senescence. The OsSWEET1b-encoded protein facilitates transmembrane glucose transport, thereby regulating intracellular sugar homeostasis [96,97]. Elevated expression of *OsSWEET1b* is significantly negatively correlated with early leaf senescence phenotypes in rice, in contrast, loss of function in *Ossweet1b* disrupts cellular energy balance and accelerates leaf senescence [96,98].

*OsFBK12* interacts with S-phase kinase-associated protein 1-like proteins and OsSAMS1 to regulate rice leaf senescence and grain diameter [99]. F-box proteins are subunits of the SCF (SKP1–Cullin–F-box) E3 ubiquitin ligase complex; the F-box motif interacts with SKP1 at the N-terminus, while the C-terminal protein–protein interaction domain recognizes specific substrates [100]. Overexpression of *OsFBK12* delays leaf senescence, whereas knockdown lines exhibit accelerated senescence [99,101], indicating that protein degradation pathways and intracellular protein homeostasis are critical determinants of senescence progression. *OsSAMS1* is involved in nitrogen metabolism and chlorophyll biosynthesis; during premature senescence, *OsSAMS1* affects photosynthetic capacity and senescence rate by regulating protein turnover and chlorophyll degradation [99].

*OsCDC48* (cell division cycle 48) has been identified as a classic leaf senescence gene in rice. The premature senescence and death 128 (*psd128*) mutant, which carries a single base substitution in *OsCDC48* leading to a premature stop codon, exhibits impaired chloroplast development, significantly reduced photosynthetic ability and chlorophyll content, and increased reactive oxygen species accumulation [102]. Recent studies have revealed that OsCDC48 interacts with its homologue OsCDC48E, and that the C-terminal region of OsCDC48 is critical for its ATPase activity and protein–protein interactions, further expanding its regulatory network [103].

Upstream hormone and ROS signals ultimately converge on a set of transcription factors that act as central regulatory hubs, directly activating downstream SAGs. Among these, NAC and WRKY transcription factors represent the two largest and most important senescence-related TF families in plants [104].

The NAC gene family is one of the largest plant-specific transcription factor families, comprising more than 150 members in rice, many of which play central roles in leaf senescence [105,106]. NAC proteins contain a conserved N-terminal NAC domain responsible for DNA binding and a variable C-terminal transcriptional regulatory region (TRR) that functions as either a transcriptional activator or repressor [106,107]. The core functional mechanism of NAC proteins is primarily manifested in their DNA-binding specificity. Their conserved NAC domain can recognize and bind to a core cis-acting element in the promoter region of target genes, known as the NAC recognition sequence, with typical motifs being “CATGTG” or variants containing “CACG” [108]. The NAC domain can be subdivided into five subdomains (A-E). Among these, subdomains A, C, and D are primarily involved in DNA binding, while subdomains D and E mediate protein dimerization, which is crucial for forming functional transcriptional complexes [109]. Variations in key amino acids within the NAC domain among different members lead to distinct preferences for the flanking sequences of the core motif, forming the basis for their functional diversity and target gene selectivity. More critically, the activity, stability, and subcellular localization of NAC proteins are precisely regulated by multi-layered and dynamic post-translational modifications (PTMs). This constitutes the core mechanism enabling NAC proteins to rapidly and reversibly integrate upstream senescence signals [110]. Phosphorylation is one of the fastest regulatory modes. Kinases such as MAPK can rapidly modulate NAC protein activity through phosphorylation. For instance, in Arabidopsis, upon activation by upstream signals, MPK3/MPK6 kinases can phosphorylate downstream transcription factors of the ERF family, thereby swiftly altering their activity and initiating the expression of stress-responsive genes. This mechanism serves as a key paradigm for the rapid regulation of transcription factors via the MAPK signaling pathway [111]. The ubiquitin–proteasome pathway acts as a “molecular switch” controlling protein homeostasis during leaf senescence. Specific E3 ubiquitin ligases can target and degrade senescence-promoting NAC factors, preventing signal overamplification and thereby delaying senescence [112]. Furthermore, SUMOylation modulates the activity and stability of various transcription factors in plants, thereby potentially fine-tuning the function of NAC proteins during processes such as oxidative stress by influencing their transcriptional activity, protein–protein interactions, or by counteracting ubiquitin-mediated degradation [113,114]. These intricately interconnected PTM networks establish NAC proteins as precise regulatory hubs that integrate and relay signals within the senescence signaling network. Different NAC members regulate senescence through diverse molecular mechanisms. For example, *OsNAC2* promotes senescence by coordinately regulating ABA metabolism: it directly activates ABA biosynthetic genes *OsNCED3* and *OsZEP1* while repressing the ABA catabolic gene *OsABA8ox1*, thereby enhancing ABA accumulation [34]. *ONAC096* accelerates senescence by positively regulating ABA signaling through activation of *OsABI5* and its downstream target *OsEEL* [115]. ONAC054 directly binds to the promoters of senescence-related genes, including *OsABI5*, STAY-GREEN, and the chlorophyll degradation gene *OsNYC1*, thereby regulating their transcriptional activity [116]. Plants overexpressing *OsNAC054* exhibit premature leaf yellowing, whereas *nac054* knockout mutants maintain leaf greenness for a longer period [116]. Among NAC TFs, OsNAP acts as a central regulatory hub controlling rice leaf senescence. Genome-wide transcriptome analyses have revealed that OsNAP directly or indirectly regulates a large set of senescence-associated genes, with its binding motifs significantly enriched in the promoters of genes involved in chlorophyll degradation and hormone metabolism [52]. Chromatin immunoprecipitation sequencing (ChIP-seq) further confirmed that OsNAP directly binds to the promoters of key downstream targets, including the chlorophyll degradation gene OsSGR and ABA metabolism genes *OsABA8ox* and *OsNCED*, thereby integrating hormonal and developmental signals to efficiently initiate the senescence program [117,118]. *OsNAC300* promotes leaf senescence by activating *OsNAP* expression, whereas *OsAGO2* suppresses *OsNAC300* expression through promoter methylation [41]. The expression of OsNAP itself is tightly regulated by multiple endogenous hormone signals. ABA and ETH pathways enhance *OsNAP* transcription through downstream TFs such as *OsbZIPs* and *OsEIL1*, which directly bind to the *OsNAP* promoter and positively regulate senescence [34]. In contrast, CTKs indirectly repress OsNAP transcription by inhibiting the expression and activity of positive senescence regulators such as OsWRKY5, thereby delaying leaf senescence.

The WRKY transcription factor family represents another major group involved in senescence regulation in higher plants, with 109 members identified in rice. WRKY proteins contain a conserved WRKY domain characterized by the WRKYGQK heptapeptide and a C-terminal zinc finger motif (C_4–7_C_22–23_HXH or CXCX_22–23_HXH), enabling specific recognition of W-box elements (TTGACC/T) in target gene promoters [119]. The core functional mechanism of WRKY proteins is primarily manifested in their DNA-binding specificity and structural dependency. At the fine structural level, the WRKY domain can be classified into three groups (I, II, and III). Group I members contain two tandem WRKY domains (one at the N-terminus and one at the C-terminus), with only the C-terminal WRKY domain responsible for specific binding to the W-box. Groups II and III possess a single WRKY domain, with Group III featuring a distinct zinc finger motif configuration (CX_7_CX_23_HX_1_C), contributing to functional differentiation from Groups I and II [120]. The WRKYGQK heptapeptide serves as the core motif for DNA binding. Key amino acids within this sequence (Trp, Arg, Tyr) form specific interactions with the core bases of the W-box element via hydrophobic interactions and hydrogen bonds. Mutations of critical residues in the WRKYGQK sequence can completely abolish the DNA-binding ability of WRKY proteins [121]. Furthermore, the C-terminal zinc finger structure maintains the three-dimensional conformational stability of the WRKY domain by chelating Zn^2+^ ions, providing the structural scaffold necessary for the specific interaction between the WRKYGQK heptapeptide and DNA. Simultaneously, the zinc finger region constitutes a key interface for WRKY proteins to form homodimers or heterodimers. Dimerization significantly enhances the binding efficiency of WRKY proteins to target gene promoters and their transcriptional regulatory activity, which is a prerequisite for the assembly of functional transcriptional complexes [122]. Variations in non-conserved amino acids within the WRKY domain, along with differences in zinc finger configuration among different WRKY members, lead to distinct preferences for the flanking sequences of the W-box element. This forms the core structural basis for their functional diversity and selective regulation of target genes. Phosphorylation is one of the most central modifications regulating WRKY protein function. The regulation of senescence processes via the phosphorylation of WRKY transcription factors by the MAPK cascade represents a conserved mechanism. In Arabidopsis, MPK3/MPK6 can phosphorylate WRKY33 to activate the expression of stress-responsive genes [123], illustrating the general mode by which WRKY proteins rapidly respond to upstream signals through phosphorylation. E3 ubiquitin ligases precisely control senescence signaling by modulating the stability of WRKY proteins. In Arabidopsis, the E3 ligase UPL5 inhibits senescence by mediating the ubiquitination and degradation of WRKY53 [124], revealing the critical role of protein degradation in WRKY-mediated senescence regulation. Additionally, SUMOylation, as an important layer of post-translational regulation, is also involved in modulating transcription factor activity and senescence processes [125]. These intricately interconnected PTM networks establish WRKY proteins as key hubs for integrating multiple signals within the senescence signaling network. Key members such as *OsWRKY5*, *OsWRKY42*, and *OsWRKY53* positively regulate leaf senescence by binding to W-box elements in the promoters of senescence-related genes, including *OsSGR* and *OsNAP* [19,33]. Integrative analysis of transcriptomic and ChIP-seq data has established OsWRKY5 as a central hub within the WRKY regulatory network, demonstrating its direct control over a broad suite of senescence-associated genes and its role in mediating crosstalk between ABA and other hormonal pathways [33]. Notably, *OsWRKY53* is strongly induced by ABA and further suppresses the expression of the antioxidant enzyme gene *OsCAT* through a feedback mechanism, resulting in reduced ROS scavenging capacity and excessive accumulation of H_2_O_2_. This leads to oxidative damage, chlorophyll degradation, and cellular structural disruption, ultimately accelerating senescence. Plants overexpressing *OsWRKY53* exhibit premature senescence and reduced germination rates, whereas knockout lines display prolonged leaf greenness and enhanced germination; moreover, ABA fails to induce early senescence in *Oswrky53* mutants [19]. *OsWRKY53* is regulated by multiple hormonal signals, including brassinosteroid signaling, enabling it to integrate diverse endogenous and environmental cues to precisely control the timing of leaf senescence [126].

In addition to the genes described above, several other classic senescence-associated genes have been characterized in rice. The *OsS40* gene family, particularly *OsS40-1*, *OsS40-2*, *OsS40-12*, and *OsS40-14*, has been shown to play potential regulatory roles in leaf senescence. Expression profiling revealed that these *OsS40* members are significantly upregulated during natural senescence as well as under stress conditions such as darkness, nitrogen deficiency, and hormone treatments (ABA, JA, and SA). *OsS40-12* functions as a nuclear-localized protein that positively regulates leaf senescence and responds to multiple environmental cues [127].

Another important regulator is *OsELS6*, which encodes an ABERRANT LATERAL ROOT FORMATION4 (ALF4)-like protein that inhibits SCF E3 ligase activity. *OsELS6* exhibits opposite regulatory effects on leaf senescence in vitro and in vivo via the jasmonic acid pathway and interacts with *OsCDC48* to coordinate senescence progression [128]. These genes, which participate in sugar transport, protein degradation, ATPase function, and transcriptional regulation, collectively highlight the multilayered architecture of the regulatory network that governs rice leaf senescence.

These findings collectively demonstrate that rice leaf senescence is governed by a complex regulatory network integrating diverse signaling pathways, including AAA-ATPase function (*OsCDC48*), S40 family-mediated transcriptional regulation, and E3 ligase modulation (*OsELS6*), in addition to the well-established NAC and WRKY transcription factor cascades [128].

**Table 1:** Transcription factors associated with leaf senescence in rice.

Protein	Locus Number	Direct Downstream Targets	Regulation Pathway (ROS/ABA/JA/Sugar)	References
OsNAP	*LOC_Os03g21060*	*OsSGR*, *OsNYC1*, *OsNYC3*, *OsRCCR1*, *Osh36*, *Osl57*, *Osh69*, *Osl85*	ABA, JA, ROS	[82,129]
OsNAC2	*LOC_Os04g38720*	*OsSGR, OsNYC3, OsNCED3, OsZEP1*	ABA, JA, ROS	[116]
ONAC106	*LOC_Os08g33670*	*OsSGR*, *OsNYC1*, *OsNAC5*, *OsNAP*, *OsEIN3*, *OsS3H*, *OsDREB2A*, *OsLEA3*, *OsbZIP23*, *LPA1*	ABA, JA, Sugar, ROS	[130]
ONAC011 (OsY37)	*LOC_Os06g46270*	-	ABA, JA, ROS	[116]
ONAC096	*LOC_Os07g04560*	*OsSGR*, *OsPAO*, *OsNYC3*, *OsRCCR1*, *Osl85*, *Osl2*, *Osl57*, *OsNAP*, *ABI5*, *OsEEL*	ABA, JA, ROS	[117]
ONAC054	*LOC_Os03g02800*	*OsNYC1, OsABI5*	ABA, JA	[18]
OsNAC109 (OsYL3)	*LOC_Os09g38000*	*OsNAP*, *OsNYC3*, *OsEATB*, *OsAMTR1*, *OsZFP185*, *OsMPS*, *OsGA2ox3*	ABA, ROS	[131]
ONAC016	*LOC_Os01g15130*	*OsNAP*	ABA, JA	[132]
OsNAC103	*LOC_Os07g42340*	SAGs	ABA, JA, ROS, Sugar	[123]
OsRL3	*LOC_Os02g47744*	*OsSGR*, *OsNYC1*, *OsRCCR1*, *Osl2*, *Osl43*, *OsSAG12-2*, *OsRK1*, *OsRAB16C*, *OsRAB16D*	ABA, JA, ROS	[133]
OsMYB102	*LOC_Os06g43090*	*OsSGR*, *OsNYC1*, *OsABF4*, *OsNAP*, *OsCYP707A6*	ABA, JA, ROS	[134]
OsWRKY80	*LOC_Os09g30400*	-	ROS, ABA	[135]
OsWRKY23	*LOC_Os01g53260*	-	ROS, ABA	[136]
OsWRKY42	*LOC_Os02g26430*	*OsMT1d*	ROS, ABA	[31]
OsWRKY5	*LOC_Os05g04640*	CCEs, *SAGs*, *OsNAP*, *OsNAC2*, *OsNCED3*, *OsNCED4*, *OsNCED5*	ABA, JA, ROS	[31]
OsWRKY53	*LOC_Os05g48840*	*OsABA8ox1, OsABA8ox2*	ABA	[137]
OsPIL1	*LOC_Os03g56950*	*OsPORB*, *OsCAO1*, *OsGLK1*, *OsGLK2*	Sugar, ROS	[138,139]
OsMYC2	*LOC_Os10g42430*	*SAGs*.	JA, ROS	[140]
OsERF101	*LOC_Os04g32620*	*OsNAP*, *OsMYC2*, *OsJAI1*, *OsCOI1a*, *OsSGR*, *OsNYC1*, *OsNYC3*	JA, ABA, ROS	[88]
OsTZF1	*LOC_Os01g09620*	Stress-responsive genes	ABA, ROS, Sugar	[141]
OsTZF2 (OsDOS)	*LOC_Os05g10670*	-	ABA, ROS	[142]
OsS40-14	*LOC_Os03g10110*	SAGs	Sugar, ROS, ABA	[143]
Ls1	*LOC_Os01g63670*	SAGs	Sugar, ROS, ABA	[144,145]

## 4. Molecular Mechanisms of Environmentally Induced Premature Senescence

### 4.1. Abiotic Stresses Converge on a Shared ABA–ROS Signaling Hub

**Figure 3:**
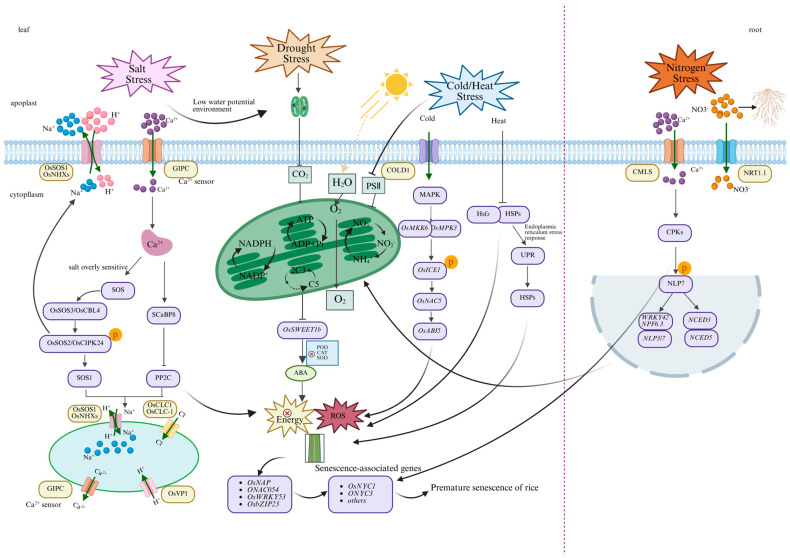
Schematic representation of stress-induced premature senescence in rice leaf and root tissues under environmental stresses. This diagram illustrates the signal perception mechanisms and induction of premature senescence in rice leaves and roots in response to salinity, drought, temperature (low/high) stress, and nitrogen deficiency. Under salt stress, ionic imbalance is sensed by plasma membrane-localized transporters and receptors, including the Na^+^/H^+^ exchanger (OsNHX) and glycosyl inositol phosphorylceramide (GIPC). These signals are transduced through the Ca^2+^–SCaBP–SOS pathway to regulate ion homeostasis. Ionic disequilibrium, together with a burst of ROS, activates senescence-associated genes and triggers premature senescence. Both salt-induced low water potential (physiological drought) and external drought stress are perceived at the plasma membrane, leading to impaired chloroplastic CO_2_ fixation and NADPH production, accompanied by enhanced abscisic acid (ABA) biosynthesis and ROS accumulation. These converging signals cooperatively activate senescence-related gene expression and drive premature senescence. Low-temperature stress is sensed by the COLD1 protein, which initiates MAPK cascade signaling, disrupts cellular metabolism, and causes ROS imbalance. High-temperature stress is perceived via HSPs and induces the unfolded protein response (UPR). Both temperature extremes promote premature senescence by activating senescence-associated genes through metabolic perturbations. Nitrogen deficiency is sensed through nitrate transporters (NRT1.1), which perceive NO_3_^−^ signals and transmit them via the CRK3–NLP signaling pathway. This signal is integrated across tissues, leading to the coordinated activation of senescence-associated genes and ultimately inducing whole-plant premature senescence. Solid arrows indicate direct signal transduction, lines with a vertical bar at the end indicate repression, and dashed arrows indicate indirect regulation through intermediate metabolites or multi-step cascades.

Rice is highly sensitive to fluctuations in water availability, salinity, and temperature. Environmental stresses such as drought, salinity, and extreme temperatures markedly affect rice growth and senescence [146]. Although these stresses differ in their physical nature, they share common cellular consequences, including disruption of water homeostasis, membrane damage, and metabolic imbalance. Ultimately, these perturbations converge on excessive ROS production and ABA accumulation, which together feed into a central pro-senescence signaling pathway [32] (Figure 3).

#### 4.1.1. Drought Stress and Premature Senescence

Drought stress disrupts water balance in rice leaves, thereby affecting multiple physiological processes. The degree of leaf dehydration is a key indicator of plant water status and reflects the severity of drought stress. Reductions in leaf water potential and cell turgor further interfere with hormone biosynthesis and transport, consequently affecting their spatial distribution within different tissues [147]. Upon perception of initial drought stimuli, plasma membrane-localized receptors convert external stress signals into intracellular chemical signals, activating downstream effectors and generating second messengers. These signals are subsequently amplified through cascade reactions, ultimately inducing extensive physiological changes.

Under drought conditions, photosynthesis is strongly inhibited. Damage to photosystem II (PSII), together with electron leakage from the photosynthetic electron transport chain, significantly enhances ROS production. Concurrently, drought rapidly induces ABA accumulation, thereby activating premature senescence-related signaling pathways [148]. ABA further exacerbates sugar accumulation in leaves by regulating the expression of the sucrose transporter *OsSWEET1b*, forming a positive feedback loop between ABA signaling and sugar accumulation. Overexpression of *JERF1* enhances drought tolerance in transgenic rice by activating the expression of two key ABA biosynthetic enzymes, *OsABA2* and *Os03G0810800*, leading to elevated ABA levels [148].

*NAC* transcription factors mitigate drought-induced damage by regulating ROS scavenging mechanisms. In rice, *SNAC1* [149] and *SNAC3* enhance drought tolerance by activating ROS-scavenging enzyme genes or repressing genes involved in ROS production [150,151]. *OsSPL10* improves drought resistance by regulating downstream *OsNAC2* expression to suppress ROS accumulation and programmed cell death [152]. *OsNAC78* positively regulates drought tolerance by maintaining ROS homeostasis through activation of *OsGSTU37*, while *OsNACIP6* interacts with *OsNAC78* and enhances its binding affinity to *OsGSTU37* [153]. WRKY transcription factors also play direct roles in drought responses by regulating downstream genes [154]. For example, *OsWRKY13* negatively regulates drought tolerance by repressing *SNAC1*, whereas *OsWRKY11* enhances drought tolerance through positive regulation of *RAB21* [155]. Additionally, *OsWRKY5* directly represses *OsMYB2*, thereby promoting ABA-induced stomatal closure and enhancing tolerance to both drought and ABA [156]. Together, these drought-responsive *NAC* and *WRKY* transcription factors illustrate how upstream signals are channeled into the ABA–ROS hub, ultimately determining the timing and severity of stress-induced premature senescence.

#### 4.1.2. Salinity Stress and Premature Senescence

Salinity stress is a major environmental factor triggering premature senescence in rice, acting through combined osmotic stress and ion toxicity to disrupt physiological metabolism and accelerate senescence. High soil salinity increases extracellular osmotic pressure, impairs root water uptake, disrupts leaf water balance, suppresses photosynthesis, and promotes excessive ROS production [157,158]. Moreover, excessive accumulation of Na^+^ disturbs intracellular ion homeostasis. In mutants deficient in the K^+^ efflux antiporter *KEA1*, salinity stress causes abnormal chloroplast morphology, loosened thylakoid stacking, reduced accumulation of photosystem-associated proteins, and disordered antioxidant enzyme activities, thereby exacerbating oxidative damage [159].

Osmotic stress and the resulting oxidative stress serve as key amplifiers of senescence progression [160]. Salt-induced low water potential creates a physiological drought environment that activates ABA signaling and induces stomatal closure [161]. While this reduces water loss, it also restricts CO_2_ supply, leading to reduced photosynthetic assimilation and increased risk of PSII photoinhibition [162]. Excess excitation energy results in massive ROS production in chloroplasts and mitochondria, while salinity stress simultaneously impairs antioxidant systems such as SOD and CAT, further weakening ROS scavenging capacity [163].

At the molecular level, salinity stress induces ABA biosynthesis and accumulation, which not only regulates stomatal behavior but also directly upregulates senescence-associated genes. Salinity stress additionally promotes ETH biosynthesis, and extensive crosstalk between ABA and ETH signaling synergistically accelerates senescence [164]. In contrast, CTK biosynthesis and transport are suppressed, leading to reduced shoot CTK levels and disruption of the dynamic balance among ABA, ETH, and CTKs. This imbalance ultimately activates downstream transcription factors, triggering the catabolism and remobilization of nutrients (proteins and nucleic acids) from leaves to grains or newly developing tissues, thereby completing the senescence program [165].

#### 4.1.3. Temperature Stress and Premature Senescence

Both high- and low-temperature stresses promote premature senescence in rice. Temperature stress accelerates senescence by enhancing ROS accumulation, reducing antioxidant enzyme activity, accelerating protein degradation, and disrupting chloroplast inner membrane structure [166].

Heat shock factors (Hsfs) are key regulators of early heat signal transduction and induce the expression of heat shock protein (HSP) genes by binding to heat shock elements (HSEs) [167]. Heat shock proteins (HSPs) function as central components of heat stress sensing by recognizing misfolded proteins and activating stress responses, including the unfolded protein response (UPR) in the endoplasmic reticulum [168]. Insufficient or imbalanced activation of this pathway leads to accumulation of misfolded proteins and aggravated oxidative stress, ultimately disrupting cellular homeostasis [153]. High-temperature stress also intensifies membrane lipid peroxidation and damages chloroplast thylakoid membranes, directly inhibiting PSII activity. Concurrently, high temperatures significantly suppress the expression of the plasma membrane hexose transporter *SWEET1b*, limiting glucose uptake and inducing sugar starvation stress [107]. Heat stress typically promotes ABA and ETH biosynthesis, forming a positive feedback loop with ROS signaling. High-temperature treatments markedly upregulate NAC transcription factors such as *OsNAP* [169,170]. NACs enhance thermotolerance by regulating heat stress-responsive genes; for example, *SNAC3* activates ROS scavenging genes and mitigates heat-induced oxidative damage [154].

Cold signals are perceived by the G-protein regulator COLD1, which activates kinase cascades such as *MEKK1–MEK2–MPK4* and modulates the phosphorylation of key kinases including *OsMPK3*. Sustained activation of MAPK cascades disrupts intracellular metabolic homeostasis, particularly electron transport during photosynthesis and respiration, leading to excessive ROS accumulation [171]. Cold stress also reduces membrane lipid fluidity, suppresses photosynthetic efficiency [172], and decreases the activities of key sugar metabolism enzymes such as fructokinase and sucrose synthase, resulting in energy deficiency. Under low-temperature conditions, ABA signaling is predominantly activated, and cold-induced signal transduction stimulates multiple transcription factor families, including *WRKYs* [88].

Both heat and cold stresses trigger ROS bursts that induce protein carbonylation, nucleic acid damage, and enzyme inactivation, generating severe oxidative stress [173,174]. In addition, sugar starvation acts as an independent signal that promotes SAG expression through activation of *OsWRKY53*, synergizing with ROS signaling to accelerate senescence.

### 4.2. Regulatory Mechanisms of Nitrogen Deficiency-Induced Senescence

Nitrogen is an essential nutrient that directly influences rice growth rate, photosynthetic efficiency, and senescence progression [17,175]. Nitrogen deficiency is a major trigger of premature senescence, acting not only by limiting chlorophyll synthesis but also by integrating nitrogen signaling, carbon–nitrogen metabolic imbalance, ROS accumulation, and hormone regulation into a coordinated senescence regulatory network [176].

Under nitrogen-deficient conditions, rice exhibits pronounced growth inhibition, including leaf yellowing, reduced chlorophyll content, and decreased photosynthetic efficiency [25]. Nitrogen limitation prevents leaves from sustaining efficient photosynthesis, restricts energy accumulation, and exacerbates oxidative damage [91]. At the signaling level, roots act as the primary nitrogen-sensing organs and transmit nitrogen deficiency signals to shoots via nitrate transporter-mediated pathways [177]. This suppresses the activity of nitrogen-responsive *NLP* transcription factors, relieving their repression of senescence-associated genes [178], while directly upregulating positive senescence regulators such as *OsNAP* (ONAC016). These factors bind to downstream SAG and chlorophyll degradation gene promoters [179], initiating chlorophyll degradation, protein catabolism, and other hallmark physiological responses of premature senescence [180,181,182].

Under low-nitrogen conditions (0 kg ha^−1^, 0 N), nitrogen-responsive transcription factors such as *OsNLP3* perceive nitrogen deficiency and activate early signaling events. This process simultaneously upregulates biosynthesis and signaling genes of senescence-promoting hormones (JA, ETH, and ABA) and induces transcription factors from the *ERF*, *WRKY*, *NAC,* and *bZIP* families (*OsERF1*, *OsWRKY42*, *OsNAP*, *OsABF4*). These transcription factors bind to promoter elements (GCC-box, W-box, ABRE) of SAGs (*SAG12*/*SAG13*) and chlorophyll degradation genes (*OsNYC1*, *OsSGR*), accelerating photosynthetic system degradation and nitrogen remobilization from leaves to grains, ultimately triggering premature senescence. A central question is how nitrogen deficiency is transduced into these transcriptional outputs; accumulating evidence points to the ABA–ROS hub as a key integrator of nitrogen-stress signals. To balance growth and stress responses, endogenous ABA levels increase markedly. Nitrogen deficiency-induced ABA biosynthesis relies on two interconnected pathways: a calcium signaling cascade triggered by inactivation of core nitrate signaling components [183] and ROS bursts driven by carbon–nitrogen metabolic imbalance [184,185].

Nitrate (NO_3_^−^) acts as a key signaling molecule under nitrogen-sufficient conditions by binding to transporters such as NRT1.1/NPF6.3 and activating the core transcription factor *NLP7* [186,187]. Activated *NLP7* translocates into the nucleus to promote nitrogen assimilation genes while repressing ABA biosynthesis. Under nitrogen deficiency, *NLP7* is rapidly inactivated and exported from the nucleus or degraded, relieving repression of ABA biosynthesis. A critical early signal is the transient elevation of cytosolic Ca^2+^, likely mediated by cyclic nucleotide-gated channels, which is decoded by calcium sensors such as calmodulin-like proteins (CMLs) and subsequently activates calcium-dependent protein kinases (CPKs) [188]. This Ca^2+^–CPK cascade rapidly converts external nitrogen signals into hormonal biosynthetic instructions [189].

Nitrogen deficiency inevitably causes carbon–nitrogen metabolic imbalance. Impaired nitrogen assimilation prevents effective utilization of carbon skeletons fixed by photosynthesis, resulting in carbon excess and over-reduction in electron transport chains, thereby triggering ROS bursts—particularly H_2_O_2_—in mitochondria, chloroplasts, and peroxisomes [190]. ROS act as crucial second messengers that both signal metabolic stress and activate stress-responsive gene expression. Under nitrogen deficiency, ROS significantly upregulate *NCED3* and *NCED5* transcription. Application of H_2_O_2_ mimics nitrogen deficiency by inducing *NCED* expression and ABA accumulation, whereas antioxidant treatment suppresses this response, linking metabolic status tightly to hormone biosynthesis [12]. These Ca^2+^ and ROS pathways interact extensively, forming a positive feedback loop that ensures robust signal amplification [191]. Ultimately, post-translational activation of NCED enzymes mediated by Ca^2+^–CPK signaling and transcriptional upregulation of *NCED* genes driven by ROS synergistically promote ABA biosynthesis [192].

At the metabolic level, nitrogen deficiency causes excessive accumulation of carbohydrates such as sucrose in leaves, which act as signaling molecules to regulate SAG expression while feedback-inhibiting key photosynthetic genes [193]. Nitrogen limitation also restricts the synthesis of antioxidants such as glutathione (GSH) and ascorbic acid (AsA), as well as related enzymes (GR, APX), reducing ROS scavenging efficiency. Excessive ROS forms positive feedback loops with nitrogen and sugar signaling [194]. At the hormonal level, ABA and ETH levels increase markedly under nitrogen deficiency, whereas anti-senescence CTKs decline due to inhibited transport from roots to shoots. Reduced CTK accumulation in leaves weakens transcriptional repression of senescence regulators such as *OsNAP*, thereby accelerating senescence [195,196].

In contrast, under optimal nitrogen conditions (240 kg ha^−1^, MN), nitrogen signaling, hormonal balance (low-level coordination between pro- and anti-senescence hormones), transcription factor expression, and metabolic processes (stable photosynthetic efficiency and coordinated nitrogen transport with grain filling) remain in an optimal state [197]. Moreover, *NAC* transcription factors enhance nitrogen use efficiency (NUE) by regulating genes involved in nitrogen uptake, transport, and utilization. For example, *SNAC1* directly binds to the promoters of nitrate transporter genes (*OsNRT2.1/2.2* and *OsNRT1.1A/1.1B*), activating their expression and promoting nitrogen uptake and utilization in rice [198]. *OsNAC68* not only responds to drought stress but also improves NUE and grain yield under nitrogen-deficient conditions [199]. Collectively, both abiotic stresses and nitrogen deficiency activate premature senescence in rice through a common framework. These conditions disrupt metabolic homeostasis, trigger ROS bursts and ABA accumulation, and engage overlapping sets of *NAC* and *WRKY* transcription factors. This convergence on the ABA–ROS signaling hub highlights a central vulnerability in rice that could be targeted to enhance stress resilience and delay premature senescence across diverse environmental challenges.

## 5. Impacts of Premature Senescence on Rice Yield

Rice yield formation depends on the photosynthetic productivity of source organs (leaves), the assimilate storage capacity of sink organs (grains), the transport efficiency of the flow system (vascular tissues), and the degree of coordination among these components, all of which are jointly regulated by endogenous and environmental factors [200]. High rice yield is achieved through highly efficient photosynthetic sources, effective assimilate transport, and strong sink capacity, supported by coordinated source–flow–sink interactions [201].

Leaves are the primary photosynthetic source organs. As critical contributors to assimilate production, leaves supply essential nutrients for plant growth and are directly linked to rice yield stability and sustainable production. Approximately 60–80% of the assimilates required for grain filling after heading originate from leaf photosynthesis. It has been estimated that extending the functional lifespan of photosynthetically active leaves by just one day during the late grain-filling stage can increase grain yield by approximately 1–2% [202]. During premature senescence, the integrity of the photosynthetic apparatus is disrupted, and chloroplasts undergo accelerated and irreversible degradation. Excessive accumulation of ROS directly damages thylakoid membranes through lipid peroxidation, accelerating the degradation of PSII reaction center proteins (D1 protein) and consequently reducing the maximum photochemical efficiency (Fv/Fm) [203]. Meanwhile, both the content and activity of ribulose-1,5-bisphosphate carboxylase/oxygenase (Rubisco), the key enzyme for photosynthetic carbon assimilation, decline prematurely. This not only limits carbon fixation in the Calvin cycle but also leads to inefficient utilization of ATP and NADPH generated during the light reactions, further aggravating photoinhibition and ROS production [202,204]. In parallel, the expression of nuclear- and chloroplast-encoded photosynthetic genes is markedly suppressed in senescing leaves, whereas SAGs are specifically activated, reflecting a metabolic shift from biosynthesis toward degradation and nutrient remobilization [205]. Ultimately, the rapid degradation of chlorophyll and proteins substantially reduces the functional photosynthetic area and the canopy active photosynthetic period (CAP), severely weakening source strength [206].

The vascular system, composed of xylem and phloem, serves as the transport pathway for assimilates from leaves to grains. The vascular bundles of the peduncle node represent the sole conduit connecting source organs to the panicle, and their structural integrity is closely associated with seed-setting rate, grain filling, and yield. Photoassimilates are transported over long distances through the phloem and unloaded into developing grains [207]. During premature senescence, excessive ROS accumulation in leaves induces membrane lipid peroxidation, directly damaging the sieve element–companion cell complex (SE–CC), leading to loss of membrane integrity and functional deterioration. Concurrently, the activities of key enzymes involved in sucrose synthesis (sucrose-phosphate synthase, SPS) and the expression of sucrose transporters (SUTs) are suppressed, resulting in reduced phloem loading efficiency [53]. In response to senescence signals, large amounts of callose are synthesized and deposited around sieve plate pores, forming dense callose plugs that physically obstruct transport channels, increase transport resistance, and significantly reduce phloem conductivity [208]. In addition, mitochondrial dysfunction during senescence limits the energy supply required to sustain pressure-flow transport. At the sink end, premature lignification and aging of peduncle vascular bundles further impair post-phloem transport, sharply reducing sucrose unloading efficiency into grains. Consequently, grain filling becomes insufficient, the proportion of unfilled grains increases, and thousand-grain weight declines significantly, ultimately leading to yield loss [209]. Thus, while source decline limits assimilate availability, vascular dysfunction further restricts their delivery to grains, compounding the impact on sink development.

The impact of premature senescence on sink organs (grains) is manifested by severely impaired grain filling and substantial yield reduction. Declines in photosynthetic capacity and vascular transport efficiency jointly reduce the flux of assimilates delivered to grains, disrupting the balance of the source–flow–sink system [210]. As strong metabolic sinks, grains rely on both sucrose supply and its efficient conversion into starch. When sucrose availability is limited, the activities and expression levels of key enzymes involved in sucrose-to-starch metabolism, such as ADP-glucose pyrophosphorylase (AGPase) and starch synthase (SS), are significantly suppressed, resulting in reduced starch synthesis and insufficient starch granule accumulation. This directly leads to a decrease in thousand-grain weight and, due to uneven assimilate distribution during early grain filling, causes filling failure or premature termination in inferior spikelets, thereby increasing grain sterility [211]. Thus, premature senescence fundamentally weakens sink activity and assimilate conversion capacity, ultimately reducing yield through decreased seed-setting rate and thousand-grain weight. The highly conserved high-yield gene *HPY1* directly binds to the promoters of Rubisco small subunit genes (*RbcS2*, *RbcS3*, and *RbcS4*) and grain-size-related genes (*CCP1* and *FLO2*), upregulating their expression and thereby enhancing photosynthetic rate, biomass accumulation, seed size, and grain yield. This provides molecular insight into the coordination of source–sink relationships in rice [211].

## 6. Strategies for Preventing Premature Senescence

### 6.1. Genetic Breeding

In terms of allele discovery and utilization, quantitative trait locus (QTL) mapping and genome-wide association studies (GWAS) have become standard approaches for identifying superior alleles conferring resistance to premature senescence. Comparative studies between indica and japonica subspecies have shown that natural variation (insertions/deletions) in the promoter region of the japonica-type *OsSGR* gene reduces its expression, thereby delaying chlorophyll degradation and leaf senescence. Introgression of such japonica alleles into high-yielding indica cultivars through marker-assisted selection (MAS) can significantly extend the photosynthetically active duration of functional leaves during grain filling without compromising yield [21,29].

In transgenic approaches, the introduction of specific genes (*FGHIJ20KAFL*) into the rice genome followed by molecular identification using reporter genes, PCR, and molecular hybridization has enabled the selection of transgenic lines exhibiting delayed leaf senescence, improved photosynthetic efficiency, and increased single-plant yield. These studies provide effective technical routes for developing premature-senescence-resistant germplasm and breeding high-yield, stable rice varieties [212]. For instance, editing key ABA receptor genes (*OsPYL5* or *OsPYL9*) reduces plant sensitivity to ABA, thereby delaying stress-induced premature senescence under drought conditions [213]. Understanding the roles and interactions of key TFs across crops offers opportunities to improve yield, quality, and stress resistance through targeted TF regulation. Natural variation in the *OsSGR* promoter increases its inducibility and accelerates senescence, leading to shortened lifespan and reduced yield. QTL analyses have revealed genetic differences in senescence timing between indica (early-senescing) and japonica (late-senescing) subspecies, which are strongly associated with *OsSGR* promoter variation [21]. Introgression of japonica-type *OsSGR* alleles into indica cultivars effectively delays senescence, enhances photosynthetic capacity, and increases grain yield [21,29].

Among transcription factors, hormone metabolism genes, and ROS homeostasis-related genes, TFs represent the most favorable targets for genetic breeding aimed at alleviating premature senescence. TFs including *OsNAP*, *OsNACs*, and *WRKYs* act as master regulators that coordinately modulate senescence, photosynthesis, hormone signaling, and stress responses [10,18]. Modulation of a single TF can systematically affect multiple downstream pathways, thus achieving stable and comprehensive regulation of leaf senescence. Hormone metabolism genes, especially those involved in ABA, CTK, and ETH pathways, also exhibit high application potential [214]. However, their regulatory effects are often pathway-specific and may lead to pleiotropic impacts on plant growth and stress tolerance. Genes responsible for ROS homeostasis effectively alleviate oxidative damage, but usually exert limited effects on delaying developmental senescence [215].

Appropriately delaying leaf senescence does not negatively influence conventional yield-related traits such as tiller number, grain size, and seed-setting rate. On the contrary, extending the functional photosynthetic period during grain filling contributes to enhanced biomass accumulation and grain yield. Nevertheless, excessive suppression of senescence may restrict nutrient remobilization and prolong growth duration, which could present a trade-off in regions with short growing seasons. Therefore, moderate, tissue-specific, and stage-specific regulation is recommended to balance delayed premature senescence and stable high yield [216].

### 6.2. Cultivation Management

Effective mitigation and delay of premature senescence requires integrated cultivation strategies that combine precise water and fertilizer management with exogenous substance application, guided by physiological and ecological mechanisms. Studies have shown that appropriate irrigation and fertilization significantly increase grain yield and nitrogen, phosphorus, and potassium uptake across rice varieties. Optimized irrigation coupled with nitrogen management promotes soil nutrient release, enhances root activity, and facilitates water and nutrient uptake and transport to aboveground tissues. This improves transpiration rate, net photosynthetic productivity, and dry matter accumulation, thereby maximizing yield potential. Enhanced root activity also increases nutrient use efficiency for N, P, and K. Water management practices such as intermittent or moist irrigation are recommended, particularly during panicle initiation and grain filling, to ensure balanced water supply and avoid root function decline or abnormal ABA accumulation caused by premature drought or prolonged flooding. Fertilization should follow the principle of “early promotion, mid-stage control, and late supplementation.” Adequate fertilization at the tillering stage promotes tiller formation and panicle development, while increased panicle fertilizer enhances nitrogen assimilation in functional leaves during grain filling and suppresses the expression of senescence-promoting factors such as *OsNAP* [217]. Controlled water and fertilizer supply from late tillering to early panicle differentiation suppresses ineffective tillers and prevents excessive canopy density, while supplemental fertilization during panicle development and grain filling improves seed-setting rate and thousand-grain weight, thereby mitigating premature senescence [218].

Exogenous regulation strategies should follow the principle of “early maintenance, mid-stage stress resistance, and late-stage leaf protection.” Foliar application of 100 μM methyl jasmonate (MeJA) from late tillering to early panicle initiation activates antioxidant systems (SOD, POD) and HSPs, inducing systemic acquired resistance and mitigating stress-induced senescence [219]. Foliar application of 0.5–1.0 mM brassinolide (BR) from late panicle initiation to heading alleviates stress damage, maintains photosynthetic efficiency, and protects cellular membranes and chloroplast ultrastructure from oxidative injury, thereby stabilizing spikelet number and delaying the onset of early leaf yellowing [220]. During early grain filling to the milky stage, foliar application of 50 μM melatonin effectively scavenges free radicals, activates auxin and CTK signaling, and suppresses ETH and ABA biosynthesis, fundamentally delaying senescence [221]. Combined application of 50–100 mg L^−1^ 5-aminolevulinic acid (5-ALA) further promotes chlorophyll synthesis, stabilizes photosynthetic membrane structures, and enhances photosynthetic productivity during grain filling, collectively mitigating the symptoms of premature senescence [222].

## 7. Conclusions and Perspectives

Premature senescence in rice is a programmed process regulated by complex molecular networks. Environmental stresses trigger ROS bursts and hormonal signaling reprogramming, activating key transcription factors such as *OsNAP* and *OsWRKY53*, which precisely regulate downstream processes including chlorophyll degradation and protein catabolism. These events ultimately lead to exhaustion of photosynthetic sources, blockage of assimilate transport, impaired grain filling, and yield loss.

Despite substantial progress, several challenges remain. Most current studies are based on bulk tissues; future research should employ single-cell sequencing technologies to resolve cell-type-specific responses during senescence [223]. The upstream regulatory mechanisms governing key transcription factors and proteins, including phosphorylation and ubiquitination of *OsNAP*, remain poorly understood. Breaking the trade-off between prolonged leaf greenness and efficient nutrient remobilization to achieve simultaneous improvements in yield and quality represents a major breeding challenge. Moreover, translating laboratory discoveries into field performance under complex and variable environments remains the ultimate goal of future research.

Through multidisciplinary integration and systems biology approaches, in-depth dissection and precise design of rice senescence processes will provide unprecedented solutions to address global climate change and ensure food security.

## Data Availability

All data discussed are available in the cited references. No new data were generated for this study.
